# Association between long term exposure to particulate matter and incident hypertension in Spain

**DOI:** 10.1038/s41598-021-99154-7

**Published:** 2021-10-05

**Authors:** Viyey Doulatram-Gamgaram, Sergio Valdés, Cristina Maldonado-Araque, Ana Lago-Sampedro, Rocío Badía-Guillén, Eva García-Escobar, Sara García-Serrano, Marta García-Vivanco, Juan Luis Garrido, Mark Richard Theobald, Victoria Gil, Fernando Martín-Llorente, Alfonso Calle-Pascual, Elena Bordiu, Luis Castaño, Elías Delgado, Josep Franch-Nadal, F. Javier Chaves, Eduard Montanya, José Luis Galán-García, Gabriel Aguilera-Venegas, Federico Soriguer, Gemma Rojo-Martínez

**Affiliations:** 1 Department of Endocrinology and Nutrition, Hospital Regional Universitario de Málaga/Universidad de Málaga, Instituto de Investigación Biomédica de Málaga-IBIMA, Malaga, Spain; 2 Centro de Investigación Biomédica en Red de Diabetes Y Enfermedades Metabólicas Asociadas (CIBERDEM), Instituto de Salud Carlos III, Madrid, Spain; 3 Centro de Investigaciones Energéticas, Medioambientales Y Tecnológicas (CIEMAT)—División de Contaminación Atmosférica, Madrid, Spain; 4 Department of Endocrinology and Nutrition and Instituto de Investigación Sanitaria University Hospital S. Carlos (IdISSC), Department Medicine II, Universidad Complutense (UCM), Madrid, Spain; 5 Hospital Universitario Cruces, BioCruces, UPV/EHU, Barakaldo, Spain; 6 Centro de Investigación Biomédica en Red de Enfermedades Raras (CIBERER), Instituto de Salud Carlos III, Madrid, Spain; 7 Department of Endocrinology and Nutrition, Hospital Universitario Central de Asturias/University of Oviedo, Instituto de Investigación Sanitaria del Principado de Asturias (ISPA), Oviedo, Spain; 8 EAP Raval Sud, Institut Català de La Salut, Red GEDAPS, Primary Care, Unitat de Suport a la Recerca (IDIAP—Fundació Jordi Gol), Barcelona, Spain; 9 Genomic Studies and Genetic Diagnosis Unit, Fundación de Investigación del Hospital Clínico de Valencia-INCLIVA, Valencia, Spain; 10 Hospital Universitari de Bellvitge, Bellvitge Biomedical Research Institute (IDIBELL), University of Barcelona, Barcelona, Spain; 11 Department of Applied Mathematics, University of Málaga, Málaga, Spain; 12 Málaga Academy of Sciences, Málaga, Spain

**Keywords:** Hypertension, Environmental impact

## Abstract

Exposure to air particulate matter has been linked with hypertension and blood pressure levels. The metabolic risks of air pollution could vary according to the specific characteristics of each area, and has not been sufficiently evaluated in Spain. We analyzed 1103 individuals, participants in a Spanish nationwide population based cohort study (di@bet.es), who were free of hypertension at baseline (2008–2010) and completed a follow-up exam of the cohort (2016–2017). Cohort participants were assigned air pollution concentrations for particulate matter < 10 μm (PM_10_) and < 2.5 μm (PM_2.5_) during follow-up (2008–2016) obtained through modeling combined with measurements taken at air quality stations (CHIMERE chemistry-transport model). Mean and SD concentrations of PM_10_ and PM_2.5_ were 20.17 ± 3.91 μg/m^3^ and 10.83 ± 2.08 μg/m^3^ respectively. During follow-up 282 cases of incident hypertension were recorded. In the fully adjusted model, compared with the lowest quartile of PM_10,_ the multivariate weighted ORs (95% CIs) for developing hypertension with increasing PM_10_ exposures were 0.82 (0.59–1.14), 1.28 (0.93–1.78) and 1.45 (1.05–2.01) in quartile 2, 3 and 4 respectively (*p* for a trend of 0.003). The corresponding weighted ORs according to PM_2.5_ exposures were 0.80 (0.57–1.13), 1.11 (0.80–1.53) and 1.48 (1.09–2.00) (*p* for trend 0.004). For each 5-μg/m^3^ increment in PM_10_ and PM_2.5_ concentrations, the odds for incident hypertension increased 1.22 (1.06–1.41) *p* = 0.007 and 1.39 (1.07–1.81) *p* = 0.02 respectively. In conclusion, our study contributes to assessing the impact of particulate pollution on the incidence of hypertension in Spain, reinforcing the need for improving air quality as much as possible in order to decrease the risk of cardiometabolic disease in the population.

## Introduction

The World Health Organization (WHO) has identified air pollution as the largest single environmental health risk worldwide with outdoor air pollution accounting for more than 4.2 million deaths every year^1^. Particulate matter (PM) is a widespread air pollutant, consisting of a complex mixture of particles of different sizes and chemistry which are suspended in the air emitted from a range of sources. Particles with an aerodynamic diameter of less than 10 µm (PM_10_) include those inhalable particles that are sufficiently small to penetrate the respiratory tract. The fine fraction of PM_10_ includes particles with an aerodynamic diameter of less than 2.5 µm (PM_2.5_) which have a high probability of deposition in the smaller airways and alveoli. Chronic exposure to PM contributes to the risk of cardiovascular and respiratory diseases^1,2^. Particulate air pollution can impact the cardiovascular system through a number of mechanisms including endothelial dysfunction, systemic and pulmonary oxidative stress and inflammation, autonomic nervous system dysfunction and epigenetic changes^3–6^. Hypertension, as one of the most important risk factors for cardiovascular disease^7^, could also be a main mediator. Accordingly, a number of experimental and epidemiological studies have described the relationship of both short-term and long-term exposure to ambient air pollutants with hypertension and blood pressure (BP) levels^8^. However, relatively fewer studies have investigated the association between long-term exposure to PM and the incidence of hypertension prospectively^9–16^. It is noteworthy that air pollution effects could vary according to the specific air pollution mix of each area, climatic features, the genetic background, as well as the underlying lifestyle and health characteristics of the studied population^17^. In this regard, previous evidence in the Spanish population has been scarce, and limited to local studies^12,18^. The di@bet.es study provides us with the opportunity to study this phenomenon, from a nationwide population-based study perspective.

## Results

### Study sample

The study population was composed of 1103 subjects, without hypertension at baseline, followed up for a mean of 7.4 years (5.8–8.8 years). The baseline characteristics of the study sample are presented in Table 1. Mean age ranged from 18 to 83 years, the percentage of women was 63.4%. Most of the population was Caucasian. The education level, proportion of smokers, drinking behavior, adherence to Mediterranean diet, physical activity and BMI are within the expected range according to the background population. Mean annual ambient temperature ranged between 9.9 and 18.8 °C, whereas mean ambient humidity was between 57 and 79%. A comparison of included and excluded individuals based on these covariates is included in Supplementary Table S1. Residential estimates of outdoor air pollution concentrations during follow-up across the study sample were relatively low, with mean and SD concentrations of PM_10_ and PM_2.5_ of 20.17 ± 3.91 μg/m^3^ and 10.83 ± 2.08 μg/m^3^ respectively (Table 2), again concordant with the background estimates for the Spanish population.

**Table 1:** Baseline characteristics of the study population (1103 individuals without HT at baseline).

	%	Mean ± SD	Range
Age (years)		44.1 ± 12.7	18–83
Gender (male)	36.6		
Ethnicity (Caucasian)	95.9		
**Education level**			
No studies	4.3		
Basic	44.6		
High school-college	51.1		
**Med diet score**		7.9 ± 1.7	13-Feb
**Physical activity (IPAQ)**			
Low	43.8		
Medium	32.8		
High	23.4		
Currently smoking	28.3		
**Alcohol intake (servings-month)**			
< 30	76.5		
30–60	14.4		
> 60	9.1		
BMI (kg/m^2^)		26.7 ± 4.2	16.7–49.4
Systolic BP (mmHg)		119.5 ± 11.3	89.5–139.7
Diastolic BP (mmHg)		72.1 ± 7.8	47.5–89.7
Mean ambient temperature (°C)		15.2 ± 2.3	9.9–18.8
Relative humidity (%)		63.5 ± 5.3	57–79

**Table 2:** Descriptive statistics for PM_10_ (μg/m^3^) and PM_2.5_ (μg/m^3^) during follow-up (2008–2016) in the study cohort.

Pollutant	Percentile					Mean	SD	Minimum	Maximum
	5th	25th	50th	75th	95th				
PM_10_	14.57	16.95	20.00	22.79	27.55	20.17	3.91	12.21	30.18
PM_2.5_	7.92	9.31	10.77	11.79	15.63	10.83	2.08	7.25	16.49

PM10, particles with an aerodynamic diameter of less than 10 µm; PM2.5, particles with an aerodynamic diameter of less than 2.5 µm; SD, standard deviation.

### Incidence of hypertension according to PM_10_ and PM_2.5_ concentrations

During follow-up, 282 cases of incident hypertension were recorded (25.6% of the study sample). Table 3 shows the incidence rates, and crude and multivariate weighted ORs and 95% CIs for developing hypertension according to PM_10_ and PM_2.5_ concentration quartiles. As can be observed, both higher PM_10_ and PM_2.5_ concentrations were significantly associated with increased ORs of incident hypertension with a significant dose response. Multivariate weighted analysis of the data even strengthened the association. In the fully adjusted model, the highest PM_10_ exposure quartile (PM_10_ 22.80–30.18 μg/m^3^) was associated with a significant OR for developing hypertension of 1.45 (95% CIs 1.05–2.01), compared with the reference category. Accordingly, the highest PM_2.5_ exposure quartile (11.80–16.49 μg/m^3^) was associated with a significant OR for incident hypertension of 1.48 (95% CIs 1.09–2.00). For each 5-μg/m^3^ increment in PM_10_ and PM_2.5_ concentrations the odds for incident hypertension were 1.22 (1.06–1.41) *p* = 0.007 and 1.39 (1.07–1.81) *p* = 0.02 respectively.

**Table 3:** Incidence rates and crude and multivariate adjusted odd ratios for developing hypertension according to PM_10_ and PM_2.5_ concentrations during follow-up (2008–2016).

	PM 10 (μg/l)					PM 10	
	12.21–16.95	16.96–20.00	20.01–22.79	22.80–30.18	*p* for trend	Per 5-μg/m^3^ increment	*p*
Number at risk	278	280	279	266			
Number developing HT	63	61	74	84			
OR no weighting (95% CI)	1 (reference)	0.95 (0.64–1.42)	1.23 (0.84–1.81)	1.58 (1.08–2.31)	0.008	1.22 (1.02–1.45)	0.026
OR with IPW * (95% CI)	1 (reference)	0.82 (0.59–1.14)	1.28 (0.93–1.78)	1.45 (1.05–2.01)	0.003	1.22 (1.06–1.41)	0.007

	PM 2.5 (μg/l)					PM 2.5	
	7.25–9.31	9.32–10.77	10.78–11.79	11.80–16.49	*p* for trend	Per 5-μg/m3 increment	*p*
Number at risk	280	275	279	269			
Number developing HT	68	60	68	86			
OR no weighting (95% CI)	1 (reference)	0.87 (0.59–1.29)	1.00 (0.68–1.48)	1.47 (1.01–2.13)	0.032	1.36 (0.99–1.88	0.06
OR with IPW * (95% CI)	1 (reference)	0.80 (0.57–1.13)	1.11 (0.80–1.53)	1.48 (1.09–2.00)	0.004	1.39 (1.07–1.81)	0.02

CI, confidence Interval; HT, hypertension; IPAQ, international physical activity questionnaire; IPW, inverse probability weighting. MedScore, mediterranean diet score; PM_10_, particles with an aerodynamic diameter of less than 10 µm; PM_2.5_, particles with an aerodynamic diameter of less than 2.5 µm; OR, odds ratio.

*Inverse probability weighting (IPW) using as confounding variables age, gender, ethnicity, education level, MedScore, IPAQ, alcohol intake, smoking, BMI, BP levels at baseline, Ambient temperature and Humidity.

### Subgroup analysis

**Figure 1:**
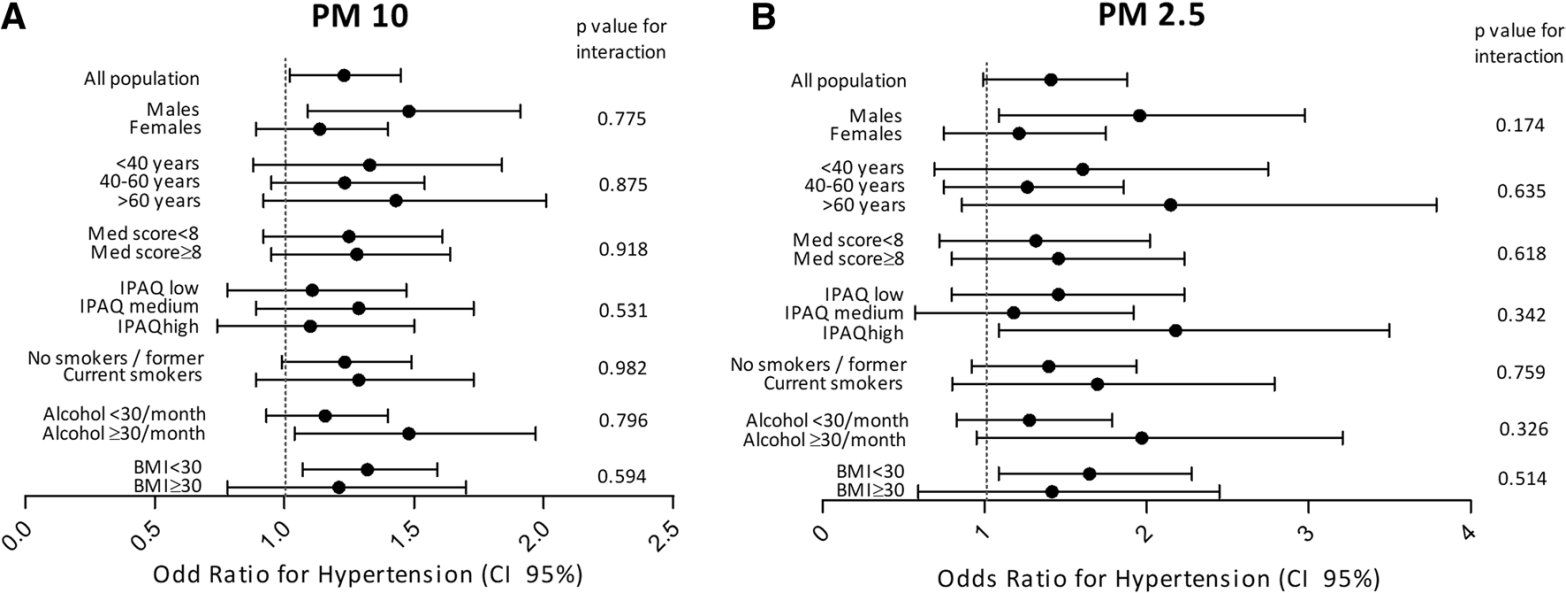
Association between PM_10_ (1**A**) and PM_2.5_ (1**B**) exposures and incident hypertension stratified by selected characteristics. Dots and bars are ORs and 95% CI for incident hypertension per 5 μg/m^3^ increment of PM concentrations of PM_10_ (1**A**) and PM_2.5_ (1**B**) and NO2 (3**C**) derived from multiple logistic regression analyses. BMI, body mass index; CI, confidence interval; IPAQ, international physical activity questionnaire; MedScore, mediterranean diet score; PM, particulate matter; PM_10_, particles with an aerodynamic diameter of less than 10 µm; PM_2.5_, particles with an aerodynamic diameter of less than 2.5 µm.

Subgroup analysis showed that the association was consistent across strata of sex, age, MedScore adherence, physical activity, smoking status, alcohol intake and BMI without any significant effect modification by these factors (Fig. 1).

## Discussion

In this nationwide cohort of Spanish non-hypertensive adults we found a positive association between PM concentrations (both PM_10_ and PM_2.5_) and the incidence of hypertension after a mean follow-up of 7.4 years. The association remained after multivariate weighted analysis of the data, showing a significant dose response, and was consistent across various subgroups.

To the best of our knowledge, this report provides the first data about the impact of air pollutants on hypertension in Spain from a National study perspective, whereas previous evidence had been limited to local studies from Northeast Spain^18^, also included in multicenter studies^12^.

Our data are consistent with a large body of evidence suggesting that air pollution may contribute to hypertension pathogenesis^8^, also supporting that the particulate component of air pollution is the most important threat for the cardiovascular system^3–6^. In this regard, although previous associations between exposure to gaseous pollutants and hypertension have shown some discrepancies, the majority of the studies reporting on long-term exposure to PM and incident hypertension have reported positive associations which are consistent with our findings^9–16^.

Interestingly, these associations have now been observed in studies from countries with relatively low concentrations of PM from North America (mean PM_2.5_ of 10.7 μg/m^3^^10^, 13.9 μg/m^3^^11^ and 13.2 μg/m^3^^13^ and Europe, (mean PM_2.5_ between 6.6 and 15 μg/m^3^^12^ and 10.8 μg/m^3^ in in the present study), and also at highly polluted regions in Asia (mean PM_2.5_ 26.5 μg/m^3^^14^, 77.7 μg/m^3^^15^ and 92.1 μg/m^3^^16^. This strongly suggests that the relation between PM exposures and hypertension development is likely a general phenomenon across different populations and over the pollution range. In fact, the increased incidence of hypertension we describe in our study occurs within PM_10_ and PM_2.5_ concentration ranges which are well below the existing European Ambient Air Quality Directive target values (PM10 < 40 µg/m^3^ and PM2.5 < 25 µg/m^3^)^19^. In contrast, our results are in line and reinforce the validity of the maximum annual concentrations for health protection suggested by the WHO (PM_10_ < 20 µg/m^3^and PM_2.5_ < 10 µg/m^3^)^20^.

The mechanism by which PM could contribute to the development of hypertension includes inflammation and oxidative stress and the triggering of autonomic nervous system imbalance affecting vascular tone and reactivity^3^. Epigenetic changes that occur during exposure may also play a role in the interaction between air pollution and Hypertension^21–23^.

Interestingly, controlled studies in humans have confirmed observational results showing that acute inhalation of concentrated particulate matter can trigger a rapid and sustained increase in blood pressure^24^.

Moreover, the use of air filtration to lower PM concentrations has demonstrated rapid effects in reducing blood pressure, further supporting this biological relationship^25–27^.

Our study has several limitations:

Firstly, our sample size is relatively small, which can affect the precision of the estimates. Due to the experimental design, the time from event until outcome was not available for analysis. We therefore used logistic regression models to calculate adjusted odds ratios, which are known to overestimate relative risks^28^.

Secondly, our food frequency questionnaire did not include data on salt consumption of the participants so that we could not adjust our analyses accordingly.

Thirdly, nationwide Spanish noise maps are also not available at present, so we could not adjust our analyses by ambient noise. Nevertheless, although long‐term noise exposure has been linked to incident hypertension in some studies^12,29^, the results have been inconsistent^30^. Moreover the associations between PM and hypertension in both cross sectional^31–33^, and longitudinal studies^9,12^ have not changed substantially after adjustment for noise.

Finally, as in other studies, we used ambient outdoor measurements modeled at the residential addresses of the participants as a proxy for exposure to air pollution, whereas no information on time–activity patterns or on the PM concentrations indoors was available. This is however a common limitation to most studies assessing the health effects of air pollution and, in fact, air quality guidelines focus primarily on ambient (outdoor) air pollution for their recommendations^20^.

As strengths of the study we included a population-based design with BP measurements of each participant at both baseline and follow-up examinations so we could identify both diagnosed and undiagnosed cases of hypertension.

We have also included extensive individual-level data including clinical, demographic and lifestyle variables which allowed us to perform a robust multivariate adjustment of the data.

Finally, our nationwide perspective, first in the Spanish population, allows us to extrapolate our results more widely than local or regional studies increasing the public health implications of the findings.

In conclusion, our study contributes to assessing the impact of particulate pollution on the incidence of hypertension in Spain. Our results reinforce the need for improving air quality as much as possible to decrease the risk of hypertension in our population, since even moderate levels such as those in this study raise the risk significantly.

## Methods

### Study design, setting and population

The di@bet.es epidemiological trial is a population based cohort study. The initial cross-sectional study was undertaken between 2008 and 2010 using a random cluster sampling to form a representative random sample of the Spanish population^34^. The study sample consisted of 5072 subjects older than 18 years, randomly selected from the National Health System registries distributed into 110 clusters (primary health care centers).

The cohort was re-evaluated in 2016–17 (follow-up time was 7.4 ± 0.5 years).

**Figure 2:**
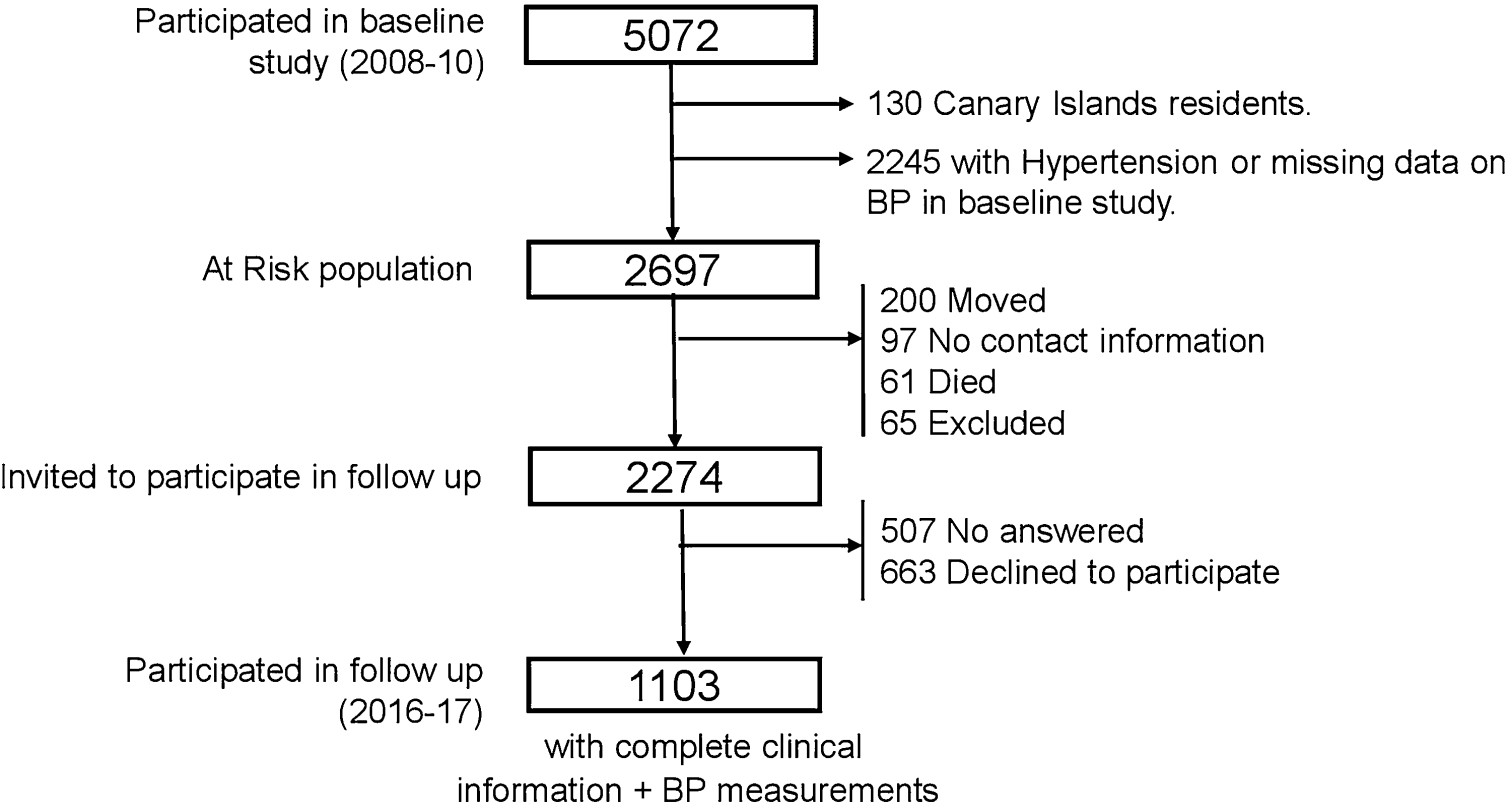
Participation flow chart.

For the present study, residents in the Canary Islands (no data on emissions available) and subjects who had hypertension or missing data on blood pressure at baseline were excluded from all the incidence calculations. Therefore, the at-risk sample included 2697 people who were residents in the Iberian Peninsula or the Balearic islands and without hypertension at baseline. Of this at-risk sample, 97 individuals had no contact information, 62 individuals had died and 200 had moved from their original location before the follow-up started. 65 other individuals were excluded (because of pregnancy or recent delivery, severe disease, institutionalized, hospitalization, or surgery during the previous month). Of the remaining 2273 individuals, 1103 subjects completed the follow-up, with complete clinical information and BP measurements available for analyses (Fig. 2). The research was carried out in accordance with the Code of Ethics of the World Medical Association (Declaration of Helsinki). Written informed consent was obtained from all the participants. The study was approved by the Ethics and Clinical Investigation Committee of the Hospital Regional Universitario de Málaga (Malaga, Spain) in addition to other regional ethics and clinical investigation committees all over Spain.

### Variables and procedures

In both phases of the study, the participants were invited to attend an examination visit at their health center with a nurse specially trained for this project. Information was collected using an interviewer administered structured questionnaire, followed by a physical examination and blood sampling.

Information on age, gender, educational level, ethnicity, smoking status, alcohol intake and other socio-demographic variables was obtained by questionnaire. Food consumption was determined by a food frequency questionnaire and adherence to the Mediterranean diet was estimated by an adaptation of a 14 item Mediterranean diet score (MedScore)^35^ Supplementary Table S2. The level of daily physical activity was estimated by the short form of the International Physical Activity Questionnaire (IPAQ)^36^. Weight and height were measured by standardized methods. The BMI was calculated. Blood pressure was measured using a blood pressure monitor (Hem-703C, Omron, Barcelona, Spain) after several minutes in a sitting position; the mean of 3 measurements taken at least 2 min apart was used for analysis. In both phases of the study, hypertension was considered if there was a previously self-reported physician-diagnosed hypertension and/or if the mean systolic blood pressure was ≥ 140 mmHg and/or the mean diastolic blood pressure was ≥ 90 mmHg^7^.

The mean annual temperature (°C) and relative humidity (%) from each site was obtained from the Spanish National Meteorological Agency website^37^.

### Exposure assessment

Mean annual PM_2.5_ and PM_10_ concentrations in Spain for the period 2008–2016 were calculated with the CHIMERE chemistry-transport model^38^ This model calculates the concentration of gaseous species and both inorganic and organic aerosols of primary and secondary origin, including primary particulate matter, mineral dust, sulphate, nitrate, ammonium, secondary organic species and water. This model has been broadly evaluated in Spain by comparison with measured air pollutants at a large set of monitoring sites^39,40^. The model was applied to a domain covering the Iberian Peninsula at a horizontal resolution of 0.1 × 0.1° (approximately 10 × 10 km^2^), except for 2015 and 2016, when a resolution of approximately 5 × 5 km^2^ was used. The modeled concentrations were corrected with observed values, by considering a methodology described by Martín et al.^41^ in which (1) a bias is calculated with respect to the observations in the Spanish air quality network of monitoring sites, (2) these biases are spatially interpolated using a krigging methodology to obtain a gridded bias, and (3) this gridded bias is applied to the modeled concentration grid. This methodology considers a different bias grid for rural and urban sites that are then combined and weighted by population density. This methodology is currently used to support the Spanish Ministry for Ecological Transition in the process of evaluation and information to the European Commission about the air quality in Spain^42^.

**Figure 3:**
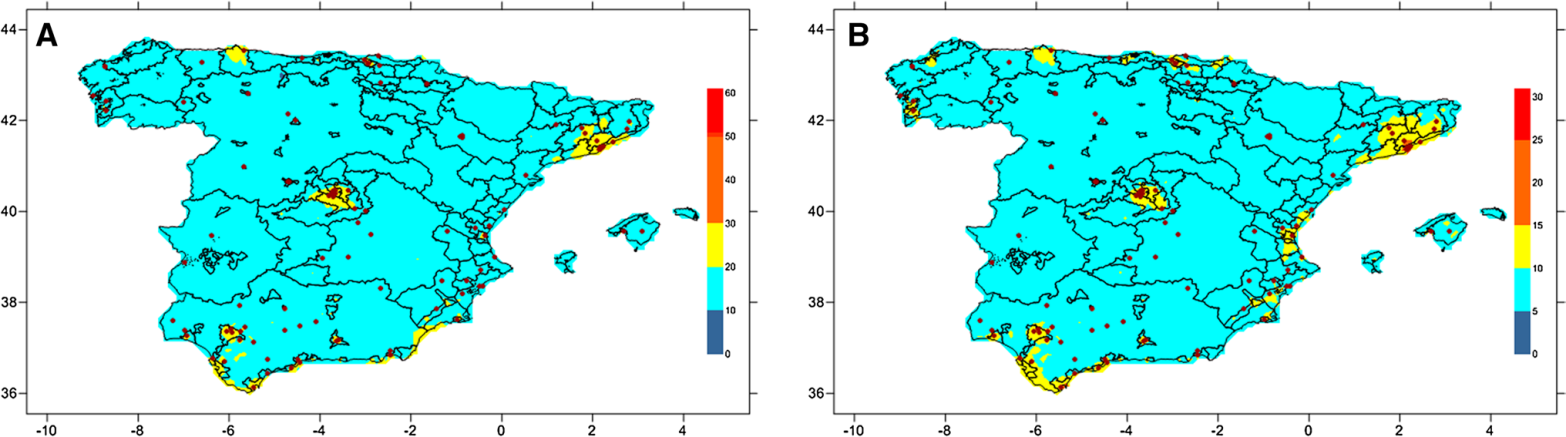
Modeled mean PM_10_ (3**A**) and PM_2.5_ (3**B**) concentrations in μg/m^3^ from 2008 to 2016 in Spain. Concentration values were calculated by applying the CHIMERE model (chim2013, https://www.lmd.polytechnique.fr/chimere/). The graphic was created with surfer (surfer 17) https://www.goldensoftware.com/products/surfer. Red dots indicate the location of clusters included in the di@bet.es study. Colour ranges are based on WHO air quality guidelines and interim targets for particulate matter^20^.

We combined the mean annual averages from each follow up year into an 8 year moving average. We assigned the 8 year exposure average at each participant address by interpolating the estimated concentrations to the centroid of their residential postal codes. Figure 3 shows the modeled concentrations of PM_10_ and PM_2.5_ during the study period in Spain, and the location of study clusters where the crossing between participant addresses and PM exposure estimates was performed.

### Statistical analysis

The study population was categorized into four groups according to the quartiles of the mean 8 year exposures of PM_10_ and PM_2.5_ during follow-up (2008–2016). Incidence rates of hypertension during follow-up were estimated on each quartile calculating the incidence rates for each 1000 inhabitant-years (95% confidence interval [CI]). We constructed logistic regression models to calculate the odds ratios for developing hypertension according to the PM_10_ and PM_2.5_ categories using the first exposure quartile as a reference. The association of each 5 μg/m^3^ increment of PM concentrations with incident hypertension was also calculated. To decrease the likelihood of bias we used an inverse probability weighting (IPW) approach by means of the propensity scores method^43^, using as possible confounding variables age, gender, ethnicity (Caucasian/others), education level (no studies/basic/high school-college), MedScore, IPAQ (low/medium/high)^36^, alcohol intake (< 30/30–60/ > 60 servings per month), smoking (never-former/current), BMI, BP levels at baseline, ambient temperature and humidity. These variables are all accepted risk factors for hypertension.

In addition, we performed subgroup analyses to test potential effect modifications in the association between PM exposures and incident hypertension by sex (male/female), age (< 40/40–60 or ≥ 60 years), MedScore (< 8 or ≥ 8), IPAQ (low, medium or high), smoking status (never-former or current), alcohol intake (< 30 or ≥ 30 per month) and BMI (< 30 or ≥ 30 kg/m^2^). Each potential modifier was examined in a separate model by adding an interaction term. We evaluated the significance of effect modification with the likelihood ratio test. Reported p values were based on two-sided tests with statistical significance set at 0.05.

## Supplementary Information

Supplementary Table S1.

Supplementary Table S2.

## Data Availability

The datasets used and/or analyzed during the current study are available from the corresponding author on reasonable request.
