# Supplementary material for: Association between long term exposure to particulate matter and incident hypertension in Spain
Source: Sci Rep. 2021 Oct 5;11:19702. doi: 10.1038/s41598-021-99154-7 (PMC8492737; doi:10.1038/s41598-021-99154-7)
Supplement: Supplementary file 1 — Supplementary Table S1. [file 41598_2021_99154_MOESM1_ESM.docx]

**Supplementary Table S1: Baseline characteristics of the study population. Comparison between participants and non-participants in follow up.**

|  |  | **Non-participants** | **Participants** | **p** |
| --- | --- | --- | --- | --- |
|  | **Number** | 1171 | 1103 |  |
|  | **Age (years)** | 40.7±13.5 | 44.1±12.7 | <0.001 |
|  | **Gender (Male)%** | 36.9 | 36.6 | 0.896 |
|  | **Ethnicity (Caucasian)** | 86.8 | 95.9 | <0.001 |
|  | **Education level (%)**  No studies  Basic  High school-college | 4.9  44.5  50.7 | 4.3  44.6  51.1 | 0.793 |
|  | **Med diet score** | 7.5±1.5 | 7.9±1.7 | <0.001 |
|  | **Physical activity (IPAQ) (%)**  Low  Medium  High | 38.7  35.3  26.0 | 43.8  32.8  23.4 | 0.042 |
|  | **Currently smoking (%)** | 36.4 | 28.3 | <0.001 |
|  | **Alcohol intake (servings-month)**  <30  30-60  >60 | 79.5  12.5  7.9 | 76.5  14.4  9.1 | 0.226 |
|  | **BMI (kg/m^2^)** | 26.2±4.5 | 26.7±4.2 | 0.008 |
|  | **Systolic BP (mmHg)** | 118.6±11.5 | 119.5±11.3 | 0.083 |
|  | **Diastolic BP (mmHg)** | 71.8±7.9 | 72.1±7.8 | 0.396 |
|  | **Mean ambient temperature (ºC)** | 15.1±2.1 | 15.2±2.3 | 0.533 |
|  | **Relative Humidity (%)** | 64.7±5.7 | 63.5±5.3 | <0.001 |

Data are mean ±SD or %
