# Supplementary material for: Association between long term exposure to particulate matter and incident hypertension in Spain
Source: Sci Rep. 2021 Oct 5;11:19702. doi: 10.1038/s41598-021-99154-7 (PMC8492737; doi:10.1038/s41598-021-99154-7)
Supplement: Supplementary file 2 — Supplementary Table S2. [file 41598_2021_99154_MOESM2_ESM.docx]

| 1 | Do you use olive oil for seasoning? | ALLWAYS= 1 point |
| --- | --- | --- |
| 2 | Do you use olive oil to cook? | ALLWAYS= 1 point |
| 3 | Do you use olive oil for frying? | ALLWAYS= 1 point |
| 4 | Vegetable servings | ≥1 per day = 1 point |
| 5 | Fruit servings | ≥3 per day = 1 point |
| 6 | Meat servings | <1 per day = 1 point |
| 7 | Butter, margarine or cream servings | <1 per day = 1 point |
| 8 | Soft drinks with sugar | <1 per day = 1 point |
| 9 | Wine servings | ≥1 per day = 1 point |
| 10 | Legume servings | ≥2 per week = 1 point |
| 11 | Fish / Seafood servings | ≥2 per week = 1 point |
| 12 | Consumption of bakery products (not homemade) | <1 per day = 1 point |
| 13 | Consumption of nuts | ≥2 per week = 1 point |
| 14 | Meat products consumption (ham, sausage) | <1 per day = 1 point |

**Supplementary Table S2 : Questionnaire of Mediterranean diet adherence**
